# A child diagnosed with midaortic syndrome and inherited thrombophilia after presenting with a stroke: A case report

**DOI:** 10.1016/j.amsu.2022.104455

**Published:** 2022-08-19

**Authors:** Narmeen Giacaman, Salem M. Tos, Mohammad G. Ibdah, Mohamad K.M. Ismail, Nael Hussein Ellahham

**Affiliations:** a Al-Quds University, College of Medicine, Palestine; b Beit-Jala Governmental Hospital, Bethlehem, Palestine; c Al-Makassed Charitable Hospital, Jerusalem, Palestine

**Keywords:** Pediatric stroke, Thrombophilia, Midaortic syndrome, Interventional cardiology

## Abstract

A 5-year-old female patient presented to the emergency room with right upper limb weakness, inability to speak and left side deviation of the mouth. Computed tomography (CT) scan was done and she was diagnosed with ischemic stroke. During her medical evaluation she was found to have high blood pressure (186/104 mm Hg). Investigations done to evaluate for secondary causes of hypertension and stroke revealed that she has inherited thrombophilia and abdominal aortic coarctation. Balloon angioplasty was done that lowered her elevated blood pressure, which was not responding well to antihypertensive medications, to near normal value for her age.

## Highlights

• MAS is a very rare condition, characterized by narrowing of distal thoracic aorta or abdominal aorta.
• Pediatric Patients can have a stroke due to a rare underlying condition.
• The patient reported had a contellation of two conditions, inherited thromophilia and MAS.

## 1 Introduction

Hypertension in children is defined as a systolic blood pressure and/or a diastolic blood pressure equal to or greater than the 95th percentile on repeated measurements. When the blood pressure falls between the 90th and 95th percentiles, it is called prehypertension [1]. Approximately 10% of Secondary hypertension in children is caused by Renovascular diseases, in which, Renal artery stenosis is considered to be the most common and most correctable cause of renovascular hypertension. Other uncommon causes of renovascular hypertension include mid-aortic syndrome (MAS) [2]. Other terms used to describe this condition include middle aortic coarctation or abdominal aortic coarctation [3].

MAS is a very rare condition that affects children and young adults, it is characterized by narrowing of distal thoracic aorta or abdominal aorta along with ostial stenosis of its major branches, especially the renal arteries [2]. It most commonly presents as severe hypertension, lower limb claudication or abdominal angina. In addition MAS patients can suffer from other complications including hypertensive encephalopathy, hypertensive heart failure and cerebrovascular accidents. Treatment options include antihypertensive medications, angioplasty and surgery [3].

Inherited thrombophilia is a genetic tendency to develop venous thromboembolism (VTE) but their role in arterial thrombosis, such as arterial ischemic stroke, remains uncertain [4]. The most common causes are the factor V Leiden and the prothrombin gene mutation G20210A, which account for about 50%–70% of the diagnosed cases. Hyperhomocysteinemia is correlated to a mildly increased risk of atherosclerosis, arterial thrombotic events and VTE. A meta-analysis showed that hyperhomocysteinemia correlates with an estimated 2.9-fold increased risk of VTE [5].

Here we report a case of a 5-year-old girl diagnosed with both inherited thrombophilia and MAS after presenting with a stroke.

This work has been reported in line with the SCARE criteria, which is used by authors, journal editors and reviewers to increases the robustness and transparency in reporting surgical cases [6].

## 2 Case presentation

A 5-year-old female patient presented to the hospital with right upper limb weakness, aphasia and left side deviation of the mouth. Right lower limb motor function was preserved. She was born at term via spontaneous vaginal delivery to 33-year-old women who received good prenatal care. The patient also had free medical and surgical history since birth.

**Fig. 1:**
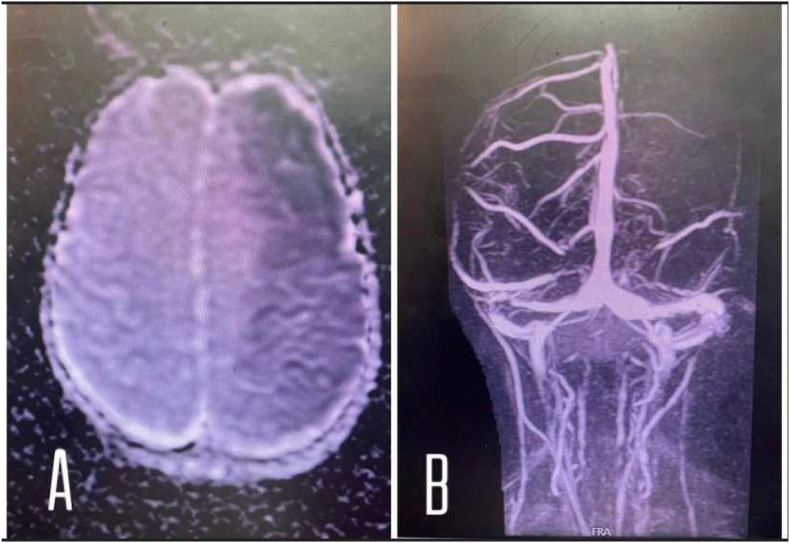
**A:** MRI shows extensive infarction of subacute aspect (ill-defined vague hypodensity), in the territory of the left middle cerebral artery without hemorrhagic transformation and no significant mass effect. **B:** MRA shows the occlusion of the left middle cerebral artery.

Acute brain stroke was suspected. Brain CT-scan without contrast was done and showed ill-defined vague hypodensity of the left parietal lobe; it showed no intracranial bleeding. Brain-MRA was done a few days later showing obstruction of the left MCA (Fig. 1). Brain-MRV and neck MRA were unremarkable.

Her vitals were normal except for the blood pressure (186/104 mmHg) for which she was started on three antihypertensive medications (amlodipine, propranolol, hydralazine) that lowered her blood pressure to (140/60 mmHg).

Further investigations were done to investigate for conditions predisposing for arterial thrombosis and secondary causes of hypertension. The thrombophilia panel (Table 1) showed that the patient is at risk of developing thrombotic events due to the heterozygous change in Factor V (FV Leiden) gene. In addition, the homozygosity of the MTHFR (A1298C) gene has been associated with 80% reduction of the MTHFR enzymatic activity leading to elevated levels of homocysteine which may result in arterial thrombosis and the homozygosity of B-Fibrinogen G544A which is described to increase the risk of thrombosis as well. She was started on enoxaparin 40 mg/day as a therapeutic dose after the stroke, then was continued on enoxaparin 20 mg/day as a prophylactic dose due to her thrombophilia profile abnormalities till this day.

**Table 1:** Thrombophilia profile.

Genetic Factor	Results
FII Prothrombin (Ala20210Gly)	Normal
MTHFR (Ala1298Cys)	Homozygous
FXIII(Val34Leu)	Normal
FV (Tyr1702Cys)	Normal
Factor V Leiden (FV Arg506Gln)	Normal
FV (His1299Arg)	Heterozygous
Factor V Cambridge (FV Arg306Thr)	Normal
MTR (Ala2756 …	Normal
MTHFR (C677T)	Normal
PAI-1 5G/4G	Heterozygous
B-Fibrinogen (Gly544Ala)	Homozygous
MTRR (Ala66Gly)	Normal

**Fig. 2:**
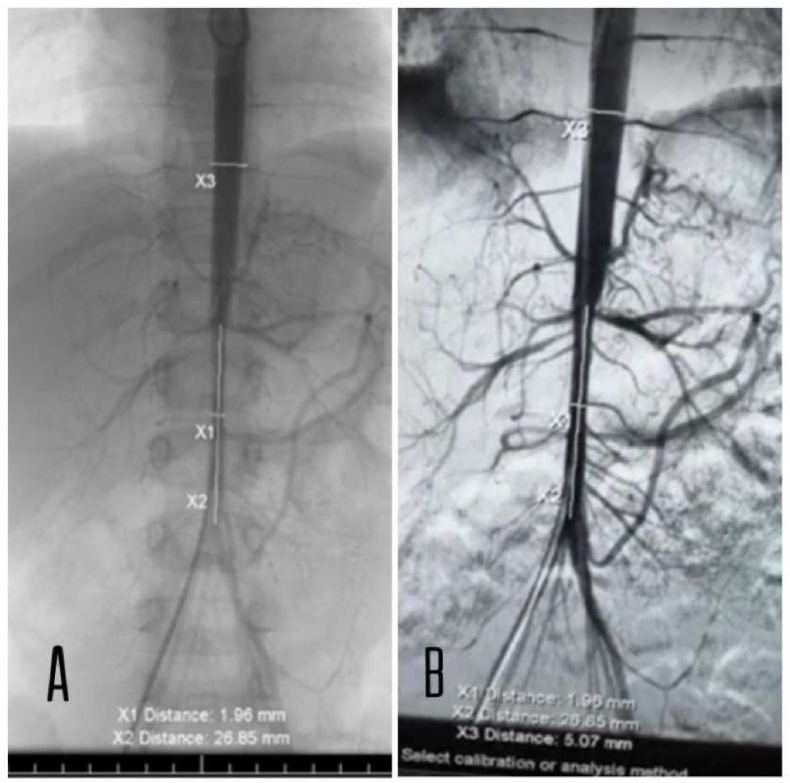
**A + B** Angiography revealed a smooth tapering of the infrarenal aorta down to the iliac bifurcation.

Angiography with I.V. contrast showed that there is decreased caliber of abdominal aorta below the level of superior mesenteric artery down to iliac arteries bifurcation (Fig. 2). Additional imaging showed that both kidneys are normal in size and position, no cysts, masses, calculi or hydronephrosis. Both renal veins are unremarkable. Preserved bilateral perinephric fat planes. Normal appearance of the liver, spleen, pancreas, and suprarenal glands. And so, she was diagnosed with Mid-aortic syndrome.

Echocardiography showed minimal mitral valve stenosis & mild regurgitation. Good global function and mild LVH. Trace pericardial effusion 4.0 mm in depth, no hemodynamic significance. No pulmonary hypertension.

**Fig. 3:**
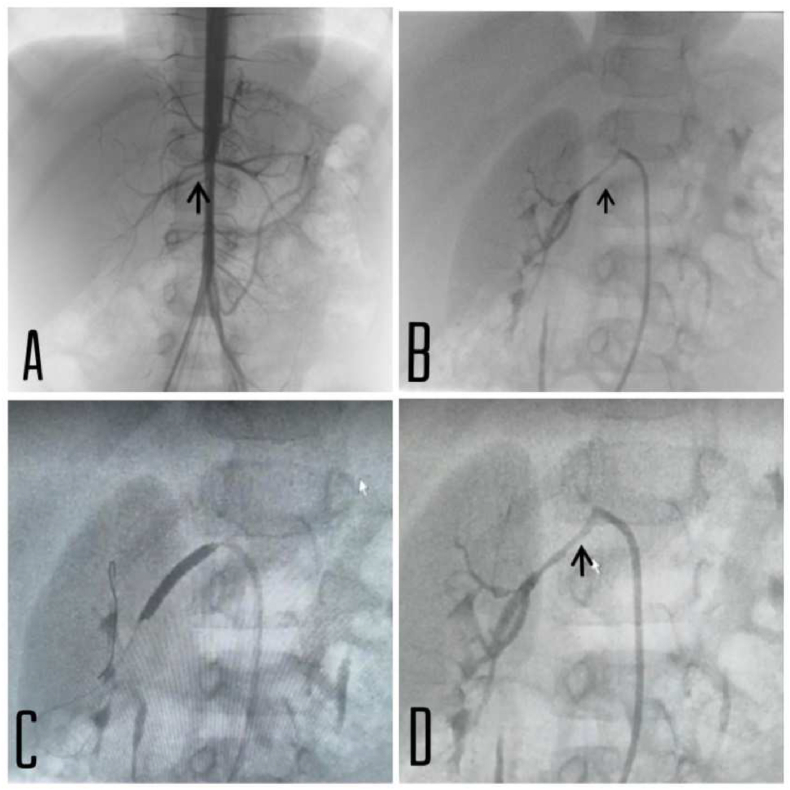
**A + B:** Angiography showed moderate right renal artery stenosis (Arrowhead) and then it bifurcate into two arteries before reaching the hilum of the right kidney. **C:** Two successful TREK RX 3*25 mm balloon dilations were advanced to the appropriate place and inflated twice until the waist disappeared in right renal artery. **D:** Renal artery post-dilatation. The left renal artery was patent (not shown in pictures).

The patient presented in this case had severe hypertension that was not well responding to the medical management thus, she was referred to an interventional pediatric cardiologist for catheterization of the renal arteries. The patient was given 1000 u heparin and the procedure was done through Right femoral artery. The catheterization showed moderate stenosis of the right renal artery, and it also bifurcates into two arteries before entering the renal hilum. Two successful TREK RX 3*25 mm balloon dilations were advanced to the appropriate place and inflated twice until the waist disappeared in right renal artery with an excellent result as the stenosis resolved (Fig. 3) and the blood pressure immediately reduced to near normal readings for her age (115/50 mmHg). The left renal artery was patent.

Pre- and post-dilatation blood pressures at ascending aorta were measured and showed significant improvement (140/96 and 115/50 respectively).

## 3 Discussion

The midaortic syndrome also known as ‘abdominal aortic coarctation’, is a rare condition that mainly affects the abdominal aorta. It is a clinical condition marked by narrowing the distal thoracic or abdominal aorta. It can be secondary to a congenital anomaly or an acquired condition. The most common site for coarctation is interrenal, then suprarenal, infrarenal and diffuse.3 the condition also involves ostial narrowing of major branches of abdominal aorta, especially renal arteries [2].

Clinical presentation depends on the grade of aortic stenosis, the extent of branch involvement and collateral formation [7].

MAS accounts for approximately 0.5–2% of all cases of aortic coarctation in general [3]; the majority of cases are idiopathic. it can be classified to congenital or acquired, congenital MAS is hypothesized to be correlated to intrauterine injuries and infections, most notably rubella, acquired cases are associated with many diseases, including aortitis, neurofibromatosis, Alagille syndrome, William syndrome, temporal and Takayasu arteritis and others [3,8]. MAS may be the sole structural abnormality or it could occur with other anomalies, including patent ductus arteriosus, bicuspid aortic valve, atrial of ventricular septal defects, renal artery stenosis, berry aneurysms in circulation of Willis [9].

Mid-aortic syndrome usually presents as arterial hypertension, which is usually unresponsive to medical therapy [8]. Although hypertension has not been identified as a risk factor in childhood stroke to date, there is preliminary evidence that suggests that hypertension may also be associated with stroke in children as in adult patients in whom hypertension is the single most important modifiable factor for stroke [10].

In Children, we usually have delayed diagnosis of MAS due to the following reasons: children are often asymptomatic, their blood pressure is not frequently measured and high values are generally dismissed as inaccurate; hence, hypertension and prehypertension are likely to be undiagnosed in the pediatric population who presented as stroke [11]. Other presenting symptoms of MAS include Headache, early fatigue on exertion, and lower-limb claudication. In addition, when mid-aortic syndrome is secondary to an acquired condition, it can be accompanied by symptoms and signs of the causal disease.

The treatment of mid-aortic syndrome can be medical, surgical, or endovascular. As conservative management carries poor prognosis, timely treatment of this syndrome is highly advised. However, before surgical intervention an attempt to achieve medical control of hypertension is still mandatory. Most patients have failed to respond to medical treatment alone, and endovascular treatment was found to have little long-term success. On contrary, surgical treatment has been shown to be curative in most patients and is therefore, despite its complexity, the treatment of choice in patients with mid-aortic syndrome [12].

A pediatric cardiologist and the interventional vascular surgeon decided to perform balloon angioplasty for this patient instead of invasive surgery. As the stenosed aortic segment is long, extending from below the level of superior mesenteric artery down to iliac arteries bifurcation. And a trial of endovascular treatment which is much less invasive than the surgery was seen as the best option regarding her case.

Endovascular treatment, primarily in the form of balloon angioplasty, has been used achieving variable success in the treatment of mid-aortic syndrome [12].

In this presented case, controlling the blood pressure by antihypertensive medications alone was poor, but the balloon angioplasty yielded an excellent result as the right two renal arteries stenosis has resolved and the blood pressure immediately reduced to near normal readings for her age.

Arterial thromboembolism is rare in children, and it is much less common than venous thrombosis in the same age group, most cases are usually complications to arterial catheterization, non-catheterization related arterial thrombosis are quite rare [13].

The incidence of stroke events, such as acute ischemic stroke, is drastically lower in pediatric population compared to adults, there is often an underlying condition associated with them, including congenital heart malformations, hemolytic anemias, collagen vascular diseases, some inborn metabolic disorders and inherited thrombophilia like antithrombin, protein C, and protein S deficiency, variants of coagulation factor V, mutations in enzymes cystathionine B synthase and MTHFR which lead to increased levels of homocysteine [14].

Hyperhomocysteinemia is considered an independent risk factor for ischemic stroke, a meta-analysis published in 2014 [15], suggests that MTHFR A1298C genetic polymorphism is associated with increased risk of ischemic stroke, moreover, the study showed stronger association with this enzyme polymorphism and ischemic stroke in Asian subgroup.

Regarding the β-fibrinogen-455G/A mutation which our patient has, Lian Gu and his coworkers [16] found that polymorphism in this gene was associated with ischemic stroke, especially in Asians but not Caucasians. This polymorphism increases fibrinogen concentration in the blood, which could accelerate the progression of atherosclerosis and early atherosclerotic plaques.

In our case, the patient presented with signs and symptoms of stroke then she was diagnosed with both inherited thrombophilia and hypertension caused by MAS. Whether the stroke in this case was caused by the inherited thrombophilia or her hypertension secondary to MAS (or both), to the best of our knowledge this is the first reported case that includes this constellation of these diseases.

## 4 Conclusion

MAS is characterized by a narrowing in the distal thoracic or abdominal aorta and considered one of the uncommon causes of hypertension in children. It usually presents with hypertension accompanied by lower limb claudication and abdominal angina, other symptoms include hypertensive encephalopathy, hypertensive heart failure and strokes. Diagnosis is frequently delayed as majority of cases are asymptomatic. Treatment of choice is surgical repair. Stroke in the pediatric population is typically caused by an underlying condition, e.g., congenital heart malformations, hemolytic anemias, collagen vascular diseases, some inborn metabolic disorders and inherited thrombophilia like antithrombin, protein C and protein S deficiency, hyperhomocysteinemia. The patient presented in this case was diagnosed with both MAS and thrombophilia after presenting with a stroke, which shows the importance of investigations in the pediatric group presenting with a stroke.

## Provenance and peer review

Not commissioned, externally peer reviewed.

## Ethical approval

The study is exempt from ethical approval in our institution.

## Sources of funding

No funding or grant support.

## Author contribution

Study concept or design: Nael Hussein Ellahham, Writing the manuscript: Narmeen Giacaman, Salem M. Tos, Mohammad G. Ibdah, Mohamad K.M. Ismail, Nael Hussein Ellahham, Review & editing the manuscript: Narmeen Giacaman, Salem M. Tos.

## Trial registry number

Not applicable.

## Guarantor

Nael Hussein Ellahham.

## Consent

Written informed consent was obtained from the patient's parents.for publication of this case report and accompanying images. A copy of the written consent is available for review by the Editor-in-Chief of this journal on request.

## Declaration of competing interest

There is no conflict of interest.
